# Characterizing physicians’ information needs at the point of care

**DOI:** 10.1007/s40037-014-0118-z

**Published:** 2014-05-28

**Authors:** Lauren A. Maggio, Olle ten Cate, Laura L. Moorhead, Feikje van Stiphout, Bianca M. R. Kramer, Edith ter Braak, Keith Posley, David Irby, Bridget C. O’Brien

**Affiliations:** 1 Stanford University School of Medicine, 300 Pasteur Drive, Room L-109, Stanford, CA 94305 USA; 2 University Medical Center Utrecht, Utrecht, The Netherlands; 3 Stanford University Graduate School of Education, Stanford, CA USA; 4 University of California, San Francisco, CA USA

**Keywords:** Evidence-based medicine, Information storage and retrieval, Medical education, Qualitative research, Internal medicine

## Abstract

Physicians have many information needs that arise at the point of care yet go unmet for a variety of reasons, including uncertainty about which information resources to select. In this study, we aimed to identify the various types of physician information needs and how these needs relate to physicians’ use of the database PubMed and the evidence summary tool UpToDate. We conducted semi-structured interviews with physicians (Stanford University, United States; n = 13; and University Medical Center Utrecht, the Netherlands; n = 9), eliciting participants’ descriptions of their information needs and related use of PubMed and/or UpToDate. Using thematic analysis, we identified six information needs: refreshing, confirming, logistics, teaching, idea generating and personal learning. Participants from both institutions similarly described their information needs and selection of resources. The identification of these six information needs and their relation to PubMed and UpToDate expands upon previously identified physician information needs and may be useful to medical educators designing evidence-based practice training for physicians.

## Introduction

Physicians have many information needs [1, 2] which, when satisfied, contribute to improved patient care outcomes [3] and physicians’ lifelong learning [4]. Information needs are an ‘expression of missing information that is required to accomplish a specific task’ [5]. Yet, many physician information needs go unmet [6] due to a variety of barriers [7], including uncertainty about which information resources to use [1, 7]. The number of information resources available to physicians is continually growing and there is a lack of guidance about which information resources to access at the point of care [8].

Fundamentally, the uncertainty surrounding information resource selection is an education problem. Researchers have suggested that knowledge of physicians’ information needs at the point of care may facilitate the development of customized training for resource selection in practice [9, 10]. To design such training, there is a need to identify practising physicians’ information needs and their relationship to information resource selection. Much of the available research on physician information needs is based on the analysis of clinical questions and classification of their types. This research has been used to create taxonomies, such as Ely’s Taxonomy of Generic Clinical Questions [11], which inform the design of informatics solutions, including the creation of question banks [6] and computerized search strategies [12]. Researchers have also examined physician information needs to inform the selection of topics for continuing medical education activities [10, 13]. While valuable, these informatics and topic-driven approaches have not provided a strategy for medical educators to utilize information needs in training programmes designed to help physicians select information resources in practice. Related to physicians’ information resource use, much of what is known focuses on identifying which resources physicians use to answer clinical questions generally [14–16]. While this provides insight into resource selection, it does not link the use of information resources to specific information needs, which have been found to shape physicians’ approaches to searches [17].

The primary aim of our research is to identify practising physicians’ information needs that can be satisfied by searching the biomedical literature. A secondary aim is to explore how information needs relate to physicians’ use of PubMed and UpToDate.

## Methods

### Design

We employed a qualitative research methodology using semi-structured interviews to capture physicians’ descriptions of their information needs in their own words.

### Context

This study was undertaken at Stanford University Medical Center (SUMC) in the US from February to March 2012 and at University Medical Center Utrecht (UMCU) in the Netherlands from August to September 2012. Both centres are research-intensive academic medical centres that offer Evidence-Based Practice (EBP) curricula and provide their physicians access to similar biomedical information resources. We selected these two medical centres because they are similar and to increase the generalizability of the findings.

Although physicians utilize a variety of information resources [18], we focused on two commonly used information resources, PubMed and UpToDate, as these are highly utilized in clinical care [15, 19]. For example, a recent study conducted using web log analysis reported that health personnel at an academic hospital accessed PubMed and UpToDate over 150,000 times in 2011 [15]. Notably other information resources such as Clin-eGuide, Five-Minute Clinical Consult and Clinical Evidence were only accessed approximately 2,000 times combined and the search engines Google and Google Scholar were similarly lightly used. Additionally, PubMed, a database, and UpToDate, an evidence summary tool, were also selected as they represent two major types of information resources. Both resources are quite different in the information they provide and their user experience. UpToDate is an evidence summary service that synthesizes available evidence on over 9,000 topics and provides a structured narrative summary [20]. PubMed is a search interface that connects users with over 23 million citations for biomedical articles and does not provide syntheses [21].

### Participants

We recruited internal medicine physicians because of the breadth of their discipline and information needs. Following approval by the SUMC human subjects review committee, one investigator (LAM), a lecturer in the Division of General Medicine Disciplines at SUMC, in consultation with a second investigator (KP), an internist, emailed 15 participants in their department an invitation to join the study. Two of the contacted internists declined to participate due to scheduling difficulties. At UMCU, following ethical approval by the Netherlands Association for Medical Education Ethical Review Board, another investigator (EtB), a professor and internist at UMCU, emailed 20 internists known to her. Nine internists scheduled interviews and 11 declined due to scheduling issues. Scheduling interviews at UMCU was difficult because the timeframe for interviews overlapped with the UMCU summer holiday season.

### Data collection

The interviewer (LAM) used the same English semi-structured interview guide (Appendix 1) for all participants no matter if they were interviewed in-person or by phone. The multidisciplinary author team designed the interview guide based on a review of the literature. We piloted the interview protocol with US and Dutch internists and made minor refinements based on their feedback. At UMCU, we also piloted the interview guide with a native Dutch-speaking internist to ensure that the use of English posed no problems.

Based on the interview guide (Appendix 1), the interviewer (LAM) first asked all participants to describe their clinical information needs. Following the description of their information needs, she requested that participants describe a clinical information need from their last day of practice and their process of satisfying that information need. Following their description, LAM specifically asked participants to indicate which of their overall clinical information needs would prompt the use of PubMed and UpToDate. All interviews were recorded and transcribed verbatim.

### Data analysis

We analyzed all transcripts using thematic analysis techniques to identify types of information needs and their relation to PubMed and UpToDate use [22]. First, two authors (LAM and BCO) familiarized themselves with the transcripts and identified passages from each transcript related to the aims of the study. Through multiple reading of the transcripts, they identified codes for participant information needs and their use of PubMed and UpToDate.

An interprofessional, multinational team (co-authors LAM, BCO, LLM, FvS, BK and KP) discussed the codes proposed by LAM and BCO and suggested additional codes based on their reading of the transcripts. After agreeing on the codes, team members coded at least four transcripts, including at least one US and one Dutch participant. Coders identified information needs and their relationship to the use of PubMed and/or UpToDate. The coders noted any cultural differences. LAM coded all transcripts and compiled all data for discussion by the coding group. Team members reached consensus on all coding. Based on the coding, LAM and BCO identified themes for information needs and PubMed and UpToDate use.

## Results

### Demographics/settings

Interviews were conducted with 22 participants (13 US, 9 Dutch) and occurred in-person (*n* = 17) or by telephone (*n* = 5). We did not detect differences in the interviewees’ responses based on telephone or in-person interview. Table 1 shows participants’ characteristics.

**Table 1:** Participant characteristics

	US participants	Dutch participants	Total Participants
Number of participants	13 (59 %)	9 (41 %)	22 (100 %)
Mean years of practice	18.53 (SD 13.97; range 3–42 years)	18.44 (SD 11.07; range 4–37 years)	18.5 (SD 12.80; range 3–42)
*Year of graduation*			
1970–1980	3 (34 %)	3 (23 %)	6 (27%)
1981–1990	2 (22 %)	2 (17 %)	4 (18 %)
1991–2000	2 (22 %)	3 (23 %)	5 (23 %)
2001–2010	2 (22 %)	5 (37 %)	7 (32 %)
Male participants	8 (62 %)	6 (66 %)	14 (64 %)
MD/PhD	0 (0 %)	9 (100 %)	9 (41 %)
Participants reporting on inpatient settings	6 (46 %)	4 (44 %)	10 (45 %)
Participants reporting on outpatient settings	7 (54 %)	5 (56 %)	12 (55 %)

We did not find differences in information needs or resource selection based on nationality. Participant information needs also did not differ on whether or not a participant possessed a PhD degree in addition to an MD degree. To offer a balance of perspectives, we included, where possible, quotes from both US (labelled S# for Stanford–US) and Dutch (labelled U# for Utrecht–Dutch) participants.

We identified six information needs: refreshing, confirming, logistics, teaching, idea generating and personal learning (Table 2). We also determined for which information needs participants selected PubMed and/or UpToDate.

**Table 2:** The six information needs identified

Reason	Definition
1. Refreshing	To update or aid in the recall of one’s own known knowledge
2. Confirming	To check one’s own knowledge for self-satisfaction or in preparation to speak, take action, advise patients, etc
	To confirm another individual’s or resource’s knowledge/coverage of a topic
3. Logistics	To answer practical questions to facilitate action
4. Teaching	To teach trainees through a variety of methods, including lecturing, role modelling, etc
5. Idea generating	To generate ideas for treatment, diagnosis or an overall sense of what is happening with a patient
6. Personal learning	To foster one’s own learning or satisfy curiosity

1. *Refreshing* To refresh their knowledge and keep current, participants utilized both UpToDate and PubMed. In a few specific instances, participants perceived PubMed as better suited to their needs for refreshing knowledge. For example, when confronted with rare conditions, several participants selected PubMed in alignment with their perception of PubMed’s relevance to ‘exotic cases’ (U6). Additionally, participants often selected PubMed when they needed more current material to refresh their knowledge, which synchronizes with the perception that PubMed is relevant for locating ‘the latest information’ (U8). Participants’ decision not to select UpToDate in this situation also aligned with participants’ uncertainty about the validity of UpToDate regarding its currency.
2. *Confirming* Participants selected both PubMed and UpToDate when seeking to confirm their own knowledge. ‘Basically I want to see if we are thinking in the same direction. To see if my thoughts are almost the same as they are in UpToDate’ (U9). In some cases, participants wanted to confirm something said by others, which typically resulted in using PubMed. For example, when hearing colleagues quote clinical trial findings, PubMed was used to locate the trials. This behaviour is consistent with participants’ frequent description of PubMed as relevant for accessing primary literature. Lastly, participants reported the need to confirm the content of UpToDate evidence summaries by searching PubMed for primary literature or utilizing the reference links available in UpToDate evidence summaries.
3. *Logistics* UpToDate was the resource primarily used when participants needed to answer logistical questions such as ‘what is the half-life of [medication xxx]?’ (S4). This links to participants’ perception of UpToDate’s content as relevant to straightforward, easy to answer, action-oriented questions. Participants’ description of the ease of using UpToDate, in contrast to the high level of effort required for PubMed, suggests one reason why PubMed is used less than UpToDate for logistical questions.
4. *Teaching* Participants role modelled both PubMed and UpToDate use for trainees, which we defined as informal teaching. One participant said: ‘I try to look up the day-to-day patients as well as patients with rare diseases when I am working with residents. We usually look up these questions when we are behind the computer together and we always use UpToDate or PubMed’ (U5). Although participants role-modelled both resources, PubMed was referenced in terms of more formal training such as ‘question-of-the-week’ activities or structured demonstrations of the PubMed interface. Participants identified their use of PubMed with trainees specifically as teaching whereas UpToDate was not labelled as such. In teaching scenarios, PubMed was often linked with EBP and several participants referenced it in the context of EBP teaching activities. Related to teaching, several participants suggested a need for additional PubMed training, guidelines for expert PubMed searching, and opportunities for feedback from PubMed experts. These requests synchronize with the perception of PubMed as difficult to use but privileged in demonstrating EBP searches.
5. *Idea generating* Participants used both PubMed and UpToDate to generate ideas. One participant said, ‘I don’t know which type of chemotherapy is used for this tumour. I have no idea.’ (U5). In this case, he consulted UpToDate, which was a common approach when participants knew the patient’s condition but needed ideas on how to proceed. This is associated with participants’ perceptions of UpToDate as relevant to straightforward, action-oriented questions. Alternatively, participants used PubMed as a clinical decision support system to figure out patient conditions for which they have little information or are ‘grasping at straws’ (S4) in terms of knowing how to proceed.
6. *Personal learning* Participants used both PubMed and UpToDate for personal learning, although PubMed was more frequently employed for this information need. This intertwines with participants’ perception of PubMed’s relevance to EBP, which is often associated with lifelong learning. In some cases, participants felt that PubMed’s perceived value as a learning tool outweighed its applicability to clinical care. Several participants stressed the importance of PubMed for medical students who are in the ‘just-learning phase’ (S11) and who are required to participate in EBP curricula. Several participants mentioned that using PubMed for learning was not realistic during patient encounters due to time constraints and was often done after work. ‘If I am trying to learn more about something then I will [use PubMed]. But I don’t think I’ve ever used [it] during a patient visit or during clinic’ (S1). This is associated with participants’ perception of PubMed as ‘challenging to use, especially for clinical care’ (S12).

Although asked to focus on patient-care questions, several participants mentioned using PubMed when undertaking research. In some cases, participants noted that PubMed was more relevant to research and less so for clinical care. ‘PubMed, I do use it all the time. But I use it for more kind of research or academic questions and a lot less for patient-care type questions’ (S1). This reasoning connects with participants’ perceptions that PubMed requires a high level of effort to use it effectively. They considered PubMed feasible in the context of research when less time pressure was felt. Participants did not mention using UpToDate for research.

Overall participants identified using both PubMed and UpToDate to satisfy each of the six information needs. In some cases, an individual mentioned using both PubMed and UpToDate to satisfy an information need. For example, several participants reported using UpToDate and PubMed in tandem when needing to confirm knowledge. In these instances, there was some uncertainty about which resource to use first to satisfy their information need.

## Discussion

Based on physicians’ descriptions we identified six information needs related to patient care: refreshing, confirming, logistics, teaching, idea generating and personal learning. In addition, participants reported using PubMed for research purposes. We have also explored for which of these information needs physicians use PubMed (a database) and/or UpToDate (an evidence-summary tool) and learned that participants used both resources to satisfy the identified information needs.

Previous studies have identified physician information needs and created taxonomies of clinical questions to help automate physicians’ access to information [9, 10]. The six identified information needs in this study align somewhat with previous research [6]. For example, Ely’s Taxonomy of Generic Clinical Questions [9, 11] identifies five broad categories including diagnosis, management, and treatment. These three broad categories could be applied to the information needs of refreshing, logistics and confirming depending on the specific details of the information need. However, the six identified information needs in this study extend Ely’s Taxonomy by adding teaching and idea generating. Furthermore, the information needs of personal learning and teaching map to the physician roles of patient care provider, lifelong learner, researcher, educator, and scholar identified by the CanMEDS Physician Competency Framework [23], which educators use in the Netherlands [24]. This alignment suggests the use of the six information needs for potentially structuring an educational approach to support physicians in the spectrum of roles that they are expected to undertake in their careers.

Medical educators have been called upon to create learning opportunities for physicians to relieve uncertainty about information resource selection [9, 10]. Approaches driven by information needs have been suggested [9–11]. We found that physicians in our study turned to different information resources for similar information needs. This finding may relate to physician’s uncertainty about resource selection and could highlight the physician’s need for more knowledge or skills to select the optimal resource for a particular information need. The six identified needs may be used to inform these educational approaches. However, future research would be needed to investigate the feasibility of using of this approach in practice and to further understand physician information needs in relation to resource selection.

Another possible interpretation of our finding that participants used both PubMed and UpToDate to satisfy similar information needs is that physicians’ information needs are complex and case specific within the context of a particular information need. For example, participants noted use of both PubMed and UpToDate for teaching, but they preferred PubMed when in formal educational settings. Similarly for idea generating participants tended to turn to UpToDate when they knew the patient’s condition, but selected PubMed when the patient’s condition was unknown. This nuanced use of information resources, which appears to depend on the context and content of physicians’ information needs (e.g. presence of trainees, patient factors, criticality of the situation, etc.), raises important issues for designing training. There may be no simple solution or algorithm for training physicians to select information resources. Instead, we may need to design training that helps physicians evaluate information resources in the context of a variety of factors such as the type of information need, features of the situation, and other people involved.

This study has several limitations. We interviewed physicians in only two countries (the US and the Netherlands), within two similar academic medical centres (Stanford and Utrecht), and within one speciality (internal medicine). Future studies might include participants from other countries and other medical specialities. This study also only focuses on two information resources, PubMed and UpToDate, which although quite popular and representative of two major types of information resources are not the only information resources physicians use. We suggest that future studies might investigate a broad range of information resources used by practising physicians, including PubMed and UpToDate, to better understand how and why physicians use these resources to satisfy each of the identified six information needs. Additionally, this study focused on physicians within academic medical centres. Future research should also examine the information needs of physicians in non-academic settings. As stated, all interviews were conducted by LAM, a medical librarian at Stanford, which had potential to bias the responses of those familiar with her role. In our analysis we did not detect any differences between the populations that suggest bias on this account.

## Conclusion

Physicians have a variety of information needs at the point of care including refreshing, confirming, logistics, teaching, idea generating and personal learning. In addition, they mentioned using PubMed for research purposes. The identification of these six information needs and their relation to PubMed and UpToDate expands upon previously identified physician information needs and sheds light on the overall complexity of physicians’ information needs. The identified information needs may be a useful starting point for designing evidence-based practice training for physicians.

## Essentials

- Physicians identified six information needs
- Information needs influence physicians selection of information resources
- The identified information needs may be a useful starting point for designing evidence-based practice training for physicians.

## Appendix 1

Interview guide:

- What information need triggers you to pose a clinical question based on a patient encounter and to search for answers in the biomedical literature?
- Please walk me through your general search strategy that you use to find information to answer your clinical question? You may find it helpful to think about your last half-day clinic or inpatient service experience and then more generally.
- We know that UpToDate is a popular resource.
  - How would you characterize UpToDate?
  - Are there any particular types of questions that lend themselves to UpToDate?
- We know that PubMed is a popular resource.
  - How would you characterize PubMed?
  - Are there any particular types of questions that lend themselves to PubMed?
